# Chemically Fueled Functionalization-Induced Self-Assembly of Block Copolymers for Transient Assemblies in Water

**DOI:** 10.1021/acspolymersau.6c00089

**Published:** 2026-06-18

**Authors:** Zehou You, Andrew J. D. Magenau

**Affiliations:** Department of Materials Science & Engineering, 6527 Drexel University, Philadelphia, Pennsylvania 19104, United States

**Keywords:** chemically fueled self-assembly, dissipative self-assembly, block copolymers, transient polymer assemblies, reaction-induced phase transitions, nonequilibrium, aqueous self-assembly

## Abstract

Chemically fueled dissipative self-assembly offers a powerful route to transient structures and biomimetic materials, yet a limited range of chemistries have been demonstrated with block copolymers to form discrete self-assembled structures. Here, we report a chemically fueled transient functionalization-induced self-assembly (ct-FISA) system in water using poly­(dimethylacrylamide-*block*-acrylic acid), p­(DMA-*b*-AA), and dimethyl sulfate (DMS) as both a functionalization reagent and chemical fuel. The water-soluble p­(DMA-*b*-AA) copolymer was synthesized by sequential reversible addition–fragmentation chain-transfer (RAFT) polymerizations and *tert*-butyl ester deprotection. Upon addition of DMS to a solution of p­(DMA-*b*-AA) in water, the carboxylate groups in the acrylic acid block were transiently methylated to form hydrophobic methyl acrylate repeat units, converting the soluble unimers into self-assembled particles. Dynamic light scattering revealed a rapid increase in particle diameter from unimers to assemblies, as large as ∼700 nm, followed by spontaneous disassembly back to unimers within approximately 70 min. Nuclear magnetic resonance confirmed transient methyl ester formation, and microscopy supported the formation of spherical particles. The particle lifetime was tunable through the initial pH, buffer concentration, and DMS loading, reflecting a balance between fuel-driven alkylation and competing fuel decomposition and ester hydrolysis. Repeated assembly–disassembly cycles were also achieved following pH readjustment. This ct-FISA platform expands the chemical toolbox for powering transient block copolymer self-assembly into discrete macromolecular assemblies in water.

## Graphical abstract

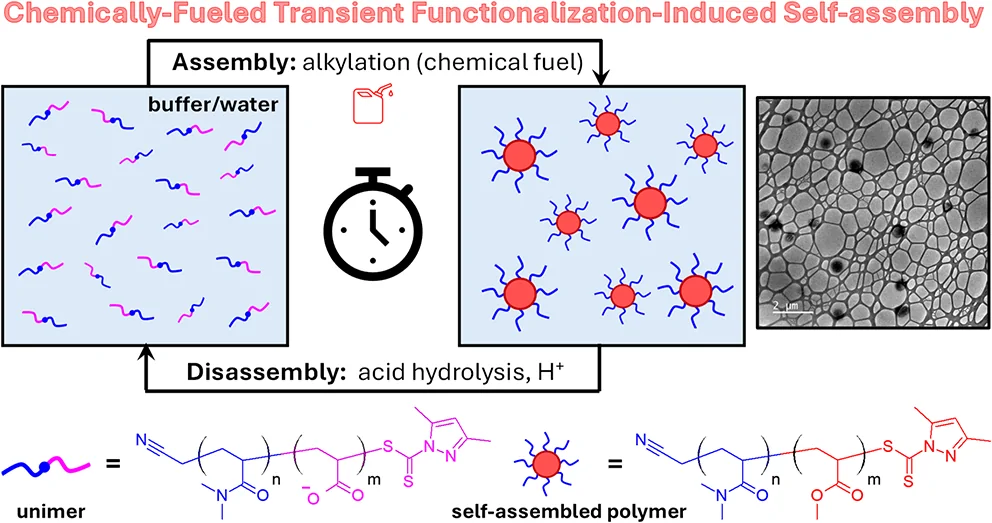

## Introduction

Reaction-induced phase transitions (RIPTs) of block copolymers have garnered increasing attention as a strategy to improve processing and access to self-assembled morphologies by coupling chemical reactivity with phase behavior.^1,2^ Broadly, RIPTs in solution refer to any phase transition (disorder-to-order or order-to-order) that is driven by a chemical reaction including polymerization, functionalization, deprotection, modification, coupling, among others.^1^ Of the solution-based RIPTs, polymerization-induced self-assembly (PISA) is the most established and a powerful process for obtaining macromolecular nanostructures, which is summarized in many excellent reviews.^3–6^ Advantageously, PISA offers scalability with fewer reaction steps and concentrated reactions up to 50%, in addition to, a diverse range of morphologies spanning traditional spheres/worms/vesicles to more complex structures.^7,8^ For morphological control in RIPTs, key parameters governing self-assembly, kinetics, and thermodynamics should be considered, e.g., block fractions, degrees of polymerization, polymer/solvent interactions, process rates; however uniquely in RIPTs, these parameters are no longer static and continuously change during the course of a reaction.^1^ By altering such parameters *in situ*, RIPTs can enable access to nonequilibrium structures that would be kinetically inaccessible or thermodynamically unstable under conventional conditions.

Functionalization induced self-assembly (FISA) is another promising, yet less explored, RIPT that operates through the functionalization, i.e., postpolymerization modification, of block copolymers (BCPs) by transforming fully soluble polymers into BCP amphiphile, thus triggering self-assembly. Transformation and amphiphilicity can be imparted to a BCP by *addition* of molar mass (functional group installation) which has been demonstrated using azo-coupling,^9^ Pd-catalyzed cross-coupling,^10^ and thiol–epoxide ring-opening.^11^ In these accounts, FISA was demonstrated to be effective with a diverse array of functional groups for obtaining tunable particle sizes in both organic and aqueous media. On the other hand, amphiphilicity can also be imparted to a BCP in FISA by a *loss* of molar mass (referred to as *deprotection*-induced or *modification*-induced self-assembly) which has been demonstrated via deacetylation,^12,13^ debenzoylation,^14^ and acidic hydrolysis of *tert*-butyl esters and aryl ethers.^15,16^ In the latter example, notably, Wang and co-workers successfully prepared a wide-range of morphologies spanning traditional spheres, worms, vesicles to more complex macromolecular structures using a single type of reaction.^15,16^ Beyond covalent functionalizations, noncovalent Lewis acid–base pairs have also been explored by Hickey et al. for pendent-group alteration to drive *in situ* self-assembly of micelles and vesicles.^17,18^ While FISA and related processes have advanced considerably, the development of *transient* systems, capable of spontaneous self-assembly and subsequent disassembly, upon an initial and singular triggering event remain largely unexplored. Such systems could provide useful model platforms for probing biologically inspired nonequilibrium behavior and for developing dynamic soft materials.

One elegant strategy to achieve structural transience can be achieved via dissipative self-assembly (DSA), where molecular organization is driven by continuous energy/fuel input, enabling structures to form and disassemble dynamically.^19–28^ Many outstanding reviews are available in the literature highlighting the impact and breadth of DSA.^23,29–31^ In a seminal publication by Boekhoven and van Esch, a chemically fueled transient self-assembly system was developed where molecular “gelators” were methylated, leading to their temporary self-assembly into fibrous structures, followed by spontaneous hydrolysis-driven disassembly.^19^ Such DSA processes can be fueled by light or other chemical reactions, where a precursor is activated into a higher-energy building block that self-assembles into a transient structure, and subsequently deactivates, reverting back to its precursor state.^1,23^ The balance between activation and deactivation rates, rather than thermodynamic stability govern structural persistence, making DSA fundamentally unique from equilibrium self-assembly and kinetic trapping. This nonequilibrium nature of DSA allows for tunable lifetimes, environmental responsiveness, and regenerative structures, offering new potential for applications in biomimetics.

Conceptually, FISA and DSA overlap, where the reactant in FISA could play two roles as both a functionalization agent for FISA and a chemical fuel for DSA. By integrating FISA with DSA, we introduce a chemically fueled transient FISA (ct-FISA) where two functionalization reactions with different forward and reverse pathways drive DSA and temporal formation of BCP assemblies (Scheme 1). Inspired by the DSA of biologically relevant gels,^19^ we instead leverage a reactive water-soluble BCP in the alkylation reaction (Scheme 1i), using dimethylsulfate (DMS, (CH_3_O)_2_SO_2_) as the chemical fuel, and hydrolysis (Scheme 1ii) to return the BCP back to its original structure through an alternate reaction pathway. To our knowledge, only carbodiimide-mediated chemistry has been demonstrated for discrete transient particles and assemblies of BCPs in solution, providing a limited chemical toolbox for transient self-assembly.^20–22^ Moreover, alkylation with DMS advantageously allows for direct modification of the carboxylate with a single reactant, reduces byproduct waste, and eliminates the need for additional coupling agents like in carbodiimide-mediated reactions. Herein, we employ poly­(*N,N*-dimethylacrylamide-*block*-acrylic acid), p­(DMA-*b*-AA), due to its pendent carboxylate groups, which readily undergo methylation to poly­(methyl acrylate) (pMA) repeat units upon reaction with DMS, inducing self-assembly (Scheme 1i). This process is reversible, as the methyl esters hydrolyze back to pAA repeat units under acidic conditions, thus triggering disassembly (Scheme 1ii). This rationally designed ct-FISA enables controllable/temporal particles and a new route to drive chemically fueled BCP self-assembly for potential applications in biomimicry and dynamic materials. We acknowledge DMS is a toxic alkylating agent and is therefore not envisioned for direct biological, biomedical, or applied smart materials applications. Rather, the presented ct-FISA system (and similar systems) may serve as model biomimetic platforms for studying underlying nonequilibrium design principles and mechanism of macromolecular organization inspired by biological activation/deactivation processes.

**Scheme 1:**
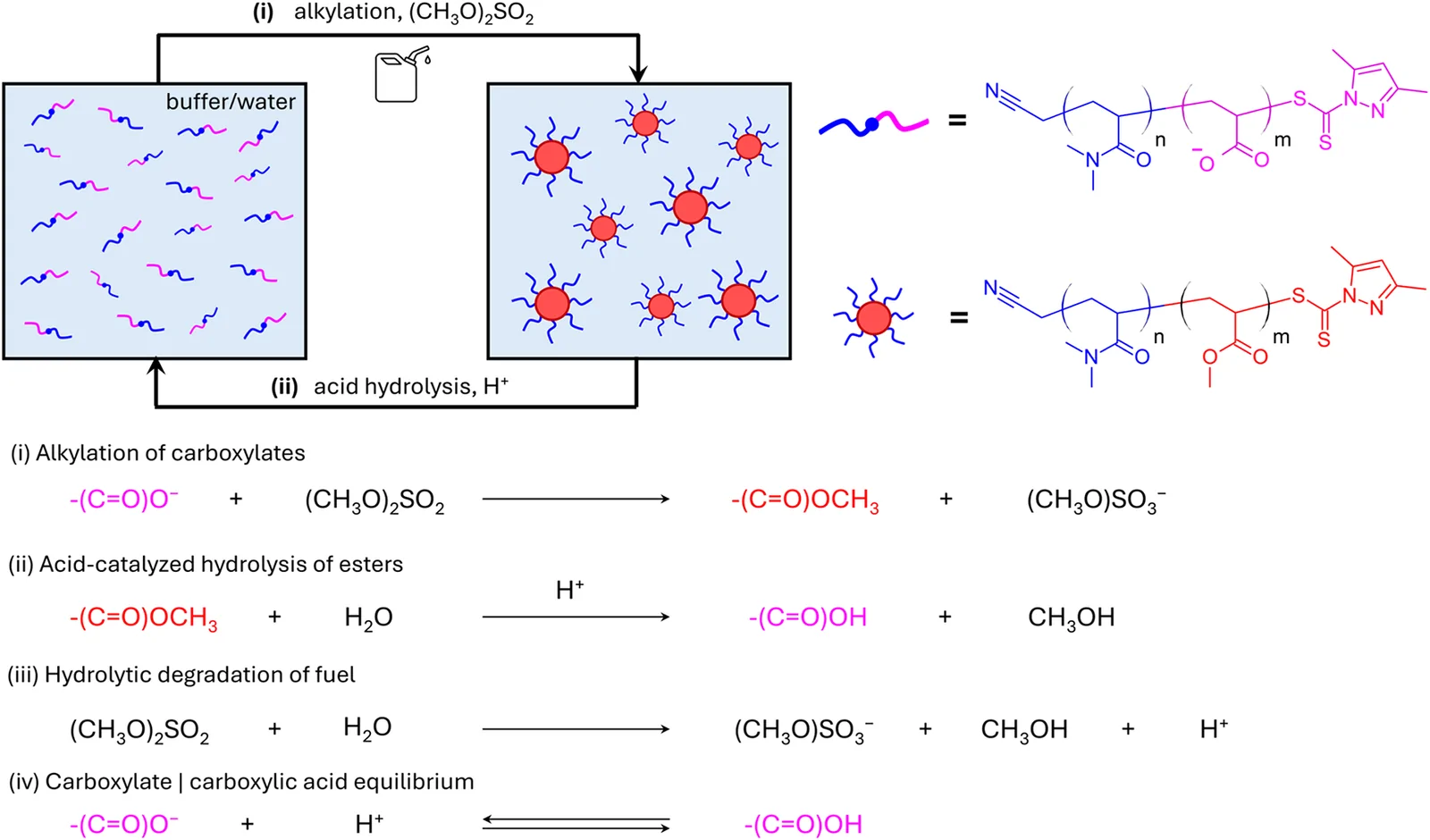
Chemically-Fueled and Transient FISA of BCPs in Water/Buffers under Ambient Conditions

## Experimental Section

### Materials

Cyanomethyl (3,5-dimethyl-1H-pyrazole)-carbodithioate (CTA, 95%, Sigma-Aldrich), 2,2′-azobis (2-methyl-propionitrile) (AIBN, 98%, Sigma-Aldrich), 1,3,5-trioxane (≥99%, Sigma-Aldrich), trifluoroacetic acid (TFA, >99%, TCI Chemicals), activated basic aluminum oxide (Alfa Aesar), boric acid (99.5%, Spectrum), sodium hydroxide (NaOH, ≥97.0%), aqueous hydrochloric acid (HCl, 37%), *N,N*-dimethylformamide (DMF, ≥9.8%, BDH), methanol (≥99.8%, BDH), hexanes (≥98.5%, Fisher Chemicals), ethyl ether (≥98%, BDH) stabilized, tetrahydrofuran (≥99%, BDH) stabilized, *N,N*-dimethylacetamide (≥99.5%, BDH), dichloromethane (DCM, ≥99.8%, BTC), water ASTM Type II (BDH), d-chloroform 99.6 atom % (TCI Chemicals), and d-dimethyl sulfoxide 99.95 atom % (d-DMSO, BDH) are used as received. *N*,*N*-dimethylacrylamide (DMA, >99%, TCI Chemicals) and *tert*-butyl acrylate (*t*BA, >98%, TCI Chemicals) were filtered through an activated, basic alumina column to remove inhibitor. Dimethyl sulfate (≥99.5%, Sigma-Aldrich) was stored in the glovebox before use. TEM grids were Ultrathin Carbon Film on Lacey Carbon Support Film (400 mesh, Copper) from Tedpella.

### Instrumentation and Methods

#### Proton Nuclear Magnetic Resonance (^1^H NMR)

For monomer conversion calculations, ^1^H NMR samples were prepared by dissolving approximately two drops (∼50 mg) of the crude reaction mixture in 1 mL of CDCl_3_. Conversion was calculated by using the areas of vinyl resonances and trioxane as an internal standard. For pure polymer analysis, ∼50 mg of the purified polymer was dissolved in 1 mL of CDCl_3_. ^1^H NMR spectra were recorded at room temperature on a Unity Inova-500 MHz spectrometer (Varian) using 64 scans and a relaxation delay of 1 s.

#### Size Exclusion Chromatography (SEC)

SEC samples were prepared by dissolving approximately one drop (∼25 mg) of the crude reaction solution in 1 mL of dimethylacetamide. The solution was vortexed to ensure dissolution and then filtered through a 0.2 μm hydrophilic PTFE filter into a 2 mL SEC vial. SEC analysis was performed using a Shimadzu LC20AD HPLC pump, equipped with a Shimadzu RID-20A refractive index detector. The polymer samples were separated using HPLC-grade dimethylacetamide as the mobile phase at a flow rate of 1 mL/min at 55 °C, with two PLgel mixed-B Agilent columns connected in series. The system was calibrated with 10 linear poly­(methyl methacrylate) standards with molecular weights ranging from 800 to 2,570,000 g/mol. SEC analysis was used to determine the number-average molecular weight (*M*_n_) and dispersity (*Đ*) of synthesized polymers.

#### Dynamic Light Scattering (DLS)

DLS samples were prepared by transferring the active reaction mixture at designated time points into a quartz cuvette for DLS analysis. Hydrodynamic diameters values were measured at 25 °C using a Malvern Zetasizer Nano ZS, equipped with a 632 nm He–Ne laser and a 173° backscattering configuration, capable of measuring particle sizes from 0.3 nm to 10 μm. The DLS results were reported as number-average diameters. Each data point represents the average of at least three measurements, with each measurement lasting 60 s.

#### Fourier-Transform Infrared (FTIR) Spectroscopy

FTIR samples were prepared by dissolving 50 mg of polymer in 0.5 mL of methanol. A 1.5 μL aliquot of the solution was transferred onto the attenuated total reflectance crystal of the FTIR instrument using a micropipette. The sample was allowed to dry under a gentle air stream for ∼10 min to ensure complete evaporation of the solvent. Infrared spectra were collected from 4000 to 500 cm^–1^ using a Bruker FTIR spectrometer equipped with an attenuated total reflection setup. Each spectrum was acquired at a spectral resolution of 4 cm^–1^, averaging over 16 scans.

#### Transmission Electron Microscopy (TEM)

TEM samples of the active material were prepared by depositing a drop (∼20 mg) of the solution onto a TEM grid. The sample was left to dry for approximately 2 min. Afterward, the sample was gently blotted using a Kim-wipe. The grids were held under vacuum at room temperature for 5 min before analysis. TEM imaging was performed on a JEOL 2100F FEG TEM microscope at an accelerating voltage of 200 kV.

#### pH Meter

pH values were measured by Mettler Toledo F20 benchtop pH meter calibrated with three buffers at pH = 4, 7, and 10.

### Syntheses

#### Reversible Addition–Fragmentation Chain-Transfer (RAFT) Polymerization of pDMA

In a 20 mL scintillation vial, CTA (30 mg, 0.14 mmol), DMA (1.3 g, 13.6 mmol), AIBN (1.2 mg, 7.1 μmol), and trioxane (0.16 g, 1.8 mmol) were dissolved in 1 g of DMF. The resulting solution was transferred to a 10 mL Schlenk flask and gently stirred for 1 min. An aliquot was then withdrawn for analysis by ^1^H NMR to determine monomer conversion. The solution was subsequently sparged with nitrogen for 40 min while stirring to remove oxygen. Once deoxygenation was complete, the flask was sealed and immersed in an oil bath preheated to 80 °C. The polymerization reaction was allowed to proceed for 3 h. After this time, the solution was exposed to air to terminate the reaction. Two additional aliquots were taken for ^1^H NMR to determine the final monomer conversion and for SEC to determine the *M*_n_ and *Đ*. The pDMA was purified by precipitation three times from DMF into diethyl ether yielding a pasty brown product.

#### Chain Extension of p­(DMA) Maco-CTA into p­(DMA-*b-t*BA)

p­(DMA) macro-CTA (0.6 g, 0.06 mmol) was dissolved in DMF (0.5 g) within a 20 mL scintillation vial. AIBN (0.5 mg, 3.11 μmol) and trioxane (0.16 g, 1.8 mmol) were added to the solution. The mixture was then transferred into a 10 mL Schlenk flask. While stirring, *t*BA (0.4 g, 3.05 mmol) was added dropwise. An aliquot was withdrawn for ^1^H NMR to assess monomer conversion. The solution was then sparged with nitrogen for 40 min while being stirred. After deoxygenation, the flask was immersed in an oil bath set to 80 °C, and the polymerization reaction was allowed to proceed for 1.5 h. The reaction was terminated by exposure to air, and two aliquots were taken for ^1^H NMR and SEC to determine the monomer conversion, *M*_n_, and *Đ*. The final p­(DMA-*b-t*BA) was purified by precipitation three times from DMF into hexane, yielding a yellow pasty product.

#### Deblocking p­(DMA-*b-t*BA) into p­(DMA-*b*-AA)

p­(DMA-*b*-tBA) (0.6 g, 43.3 μmol) was dissolved in 1.3 g of DCM in a scintillation vial and transferred into a 10 mL Schlenk flask. TFA (1.4 g, 12.2 mmol) was added dropwise under continuous stirring. The reaction flask was then immersed in an oil bath set to 45 °C and the deblocking reaction was allowed to proceed overnight. The following day, the crude reaction mixture was subjected to rotary evaporation to remove the residual DCM and TFA. The resulting p­(DMA-*b*-AA) was last dissolved in methanol and purified by precipitation three times into diethyl ether.

#### Chemically Fueled Transient FISA

A representative ct-FISA experiment was conducted using 0.5 wt % BCP in 1 mL of 0.9 M buffer, pH = 6.4, with a [DMS]/[COOH] = 5.9. To prepare the reaction, 5 mg of pure p­(DMA_100_-*b*-AA_40_), 16.1 μmol COOH groups, was dissolved in 0.8 mL of 0.9 M pH 7 borate buffer in a 2 mL vial. The solution was vortexed for 1 h to ensure dissolution of the BCP. The pH was then adjusted to the desired initial value using 2 M NaOH or 1 M HCl and a pH meter. After pH adjustment, the solution was slightly diluted with 0.9 M pH 6.4 buffer to a final volume of 1 mL. The prepared solution was then filtered into a 7 mL scintillation vial equipped with a magnetic stirrer. Finally, DMS (12 mg, 95 μmol) was transferred from a glovebox just before use and added to the solution. The mixture was stirred for 1 min to dissolve the DMS, which initiated the chemically fueled transient self-assembly process.

## Results and Discussion

### Synthesis of p­(DMA-*b*-AA)

The final BCP, p­(DMA-*b*-AA), needed for downstream transient FISA experiments was synthesized in three steps (Figure 1a): two sequential RAFT polymerizations for molecular weight control and narrow dispersity (*Đ*) in steps I–II, followed by a deblocking reaction to achieve the desired carboxylic acid functionality in step III. The DMA block was selected because of its water solubility and inertness, whereas the pAA block was selected for its water solubility and reactivity toward DMS during alkylation. Here, an asymmetrical BCP was targeted with a long *hydrophilic* corona-forming block of DMA repeat units, and when functionalized, a short *hydrophobic* core-forming block of pMA repeat units.

**Figure 1:**
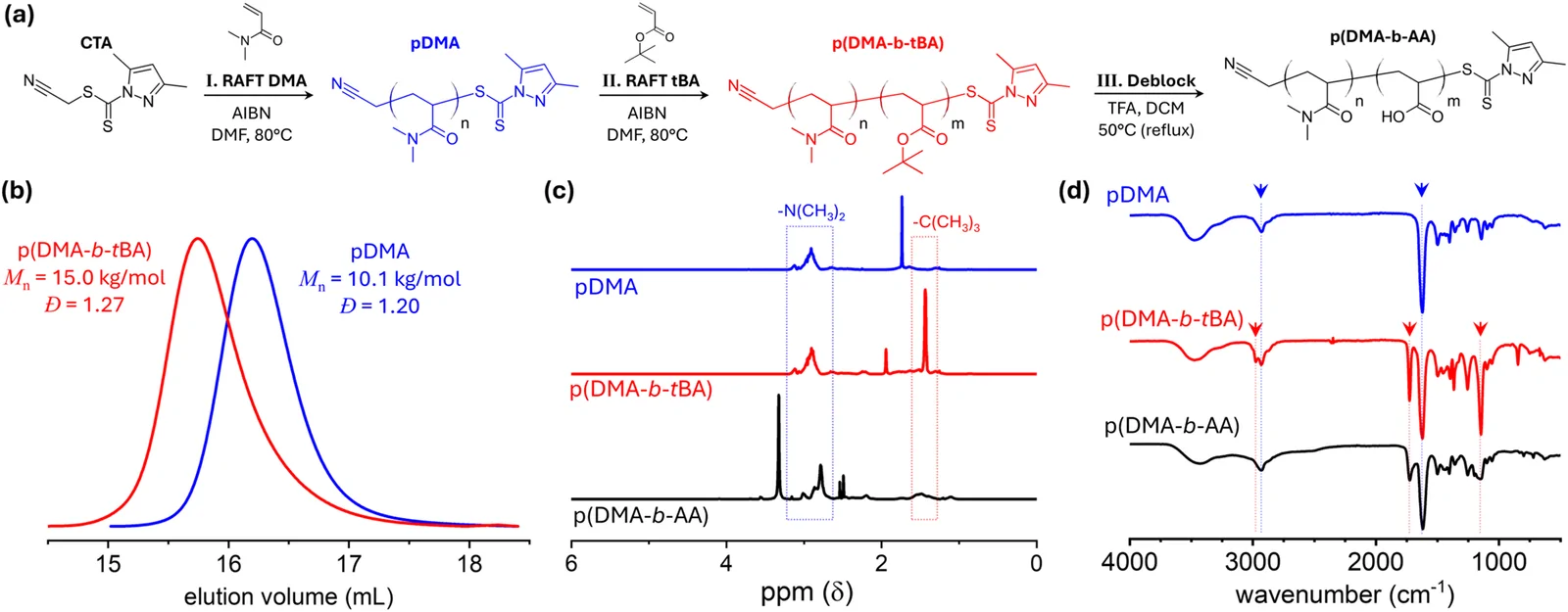
(a) Reaction scheme for synthesizing a BCP, p­(DMA-*b*-AA), for ct-FISA. (b) SEC traces confirming successful macro-CTA synthesis and chain-extension into a BCP. (c) ^1^H NMR spectra of pDMA and p­(DMA-*b-t*BA) in d-chloroform and p­(DMA-*b*-AA) in d-DMSO. (d) FTIR spectra of pDMA, p­(DMA-*b-t*BA), and p­(DMA-*b*-AA).

The first RAFT polymerization of DMA was initiated with AIBN and mediated by a dithiocarbamate chain transfer agent (CTA, Figure 1a), i.e., cyanomethyl (3,5-dimethyl-1H-pyrazole)-carbodithioate, using a molar ratio of [DMA]:[CTA]:[AIBN] ≈ 100:1:0.05. This CTA was chosen because of its amphiphilicity, ensuring that the resultant BCP would remain fully soluble in water and have a minimal impact on the self-assembly process, unlike long alkyl chain CTAs which are hydrophobic and can cause self-assembly themselves.^32^ Furthermore, the selected CTA is known to be highly effective with more activated monomers like acrylamides and acrylates as we have selected here.^33^ In first step (Figure 1a, step I), RAFT polymerization of DMA achieved ∼99% monomer conversion in just 3 h based upon ^1^H NMR using the vinyl resonances around 6.5–5.5 ppm (Figure S1). In support of NMR, SEC analysis (Figure 1b) confirmed the successful polymerization of DMA, revealing the formation of a narrow *Đ* polymer with a degree of polymerization of ∼100. The number-average molecular weight from SEC (*M*_n, SEC_ ≈ 10.1 kg/mol) was consistent with the theoretical molecular weight based upon NMR conversion (*M*_n, NMR_ ≈ 9.7 kg/mol). After purification, the pDMA structure was primarily confirmed by its characteristic methyl groups, N­(CH_3_)_2_, in ^1^H NMR at ∼3.2 ppm (Figure 1c, dashed blue box),^34,35^ along with its strong tertiary amide C=O band (1624 cm^–1^) and medium C–H stretching band (2927 cm^–1^) from FTIR (Figure 1d, blue arrows).^36,37^

Subsequently, the pDMA macro-CTA was chain-extended with *t*BA to yield p­(DMA-*b-t*BA) (Figure 1a, step II). RAFT was conducted using molar ratios of [*t*BA]:[macro-CTA]:[AIBN] = 50:1:0.05. *t*BA was chosen as the monomer because its *tert*-butyl group permits facile deprotection into carboxylic acids under acidic conditions.^38^ After RAFT polymerization, ^1^H NMR analysis confirmed the polymerization reached ∼82% conversion and SEC revealed a shift to higher molecular weight, validating chain-extension, and a moderately narrow *Đ* of 1.27 (Figure 1b). Furthermore, the SEC trace did not show any significant signs of residual macro-CTA. Together, BCP molecular weight values from NMR and SEC were in excellent agreement with the *M*_n, NMR_ ≈ 14.9 kg/mol and *M*_n, SEC_ ≈ 15.0 kg/mol, giving a degree of polymerization ≈ 40 for the *t*BA block. The chemical structure of the BCP was confirmed with ^1^H NMR from the *tert*-butyl resonances at ∼1.4 ppm (Figure 1c, red spectra)^39^ and the FTIR bands from the *tert*-butyl ester at 2975 cm^–1^ (C–H stretch), 1726 cm^–1^ (ester C=O), and 1147 cm^–1^ (ester C–O stretch) (Figure 1d, red arrows and red spectra).^40^

The final synthetic step needed to obtain the BCP involved an acid-catalyzed deblocking of p­(DMA-*b-t*BA) into p­(DMA-*b*-AA). ^1^H NMR confirmed successful deprotection via the disappearance of the *tert*-butyl resonance at ∼1.4 ppm (Figure 1c, dashed red box). FTIR further validated this transformation, showing the loss of the C–H stretch at 2975 cm^–1^ and ester C–O stretch at 1147 cm^–1^ after removal of the *tert*-butyl group (Figure 1d, red arrows). Collectively, these results confirm the successful synthesis of p­(DMA_100_-*b*-AA_40_) with degrees of polymerization of ∼100 and 40 for the pDMA and pAA blocks, respectively. Additional NMR and FTIR spectra are available in the SI for each synthetic step (Figure S2–S7)

### Transient Chemically Fueled BCP Assembly and Disassembly

After the successful synthesis of p­(DMA-*b*-AA), ct-FISA was attempted by dissolving 0.5 wt % BCP into a 0.9 M borate buffer at a pH = 6.4, followed by injection of 5.9 equiv of alkylating agent to polymeric carboxylic acids (Scheme 1, step (i)). Since the p*K*_a_ of the pAA block is ∼4.5,^41,42^ the polymeric carboxylic acids exist as carboxylates at pH = 6.4, a necessity for the alkylation reaction with DMS. Then, immediately after addition of DMS, DLS was used to monitor the particle size as a function of time to gain insights into the assembly/disassembly processes (Figure 2a,b) along with NMR and TEM for structural analysis. Additional DLS distributions (intensity, number, volume) are all available in Figure S8.

**Figure 2:**
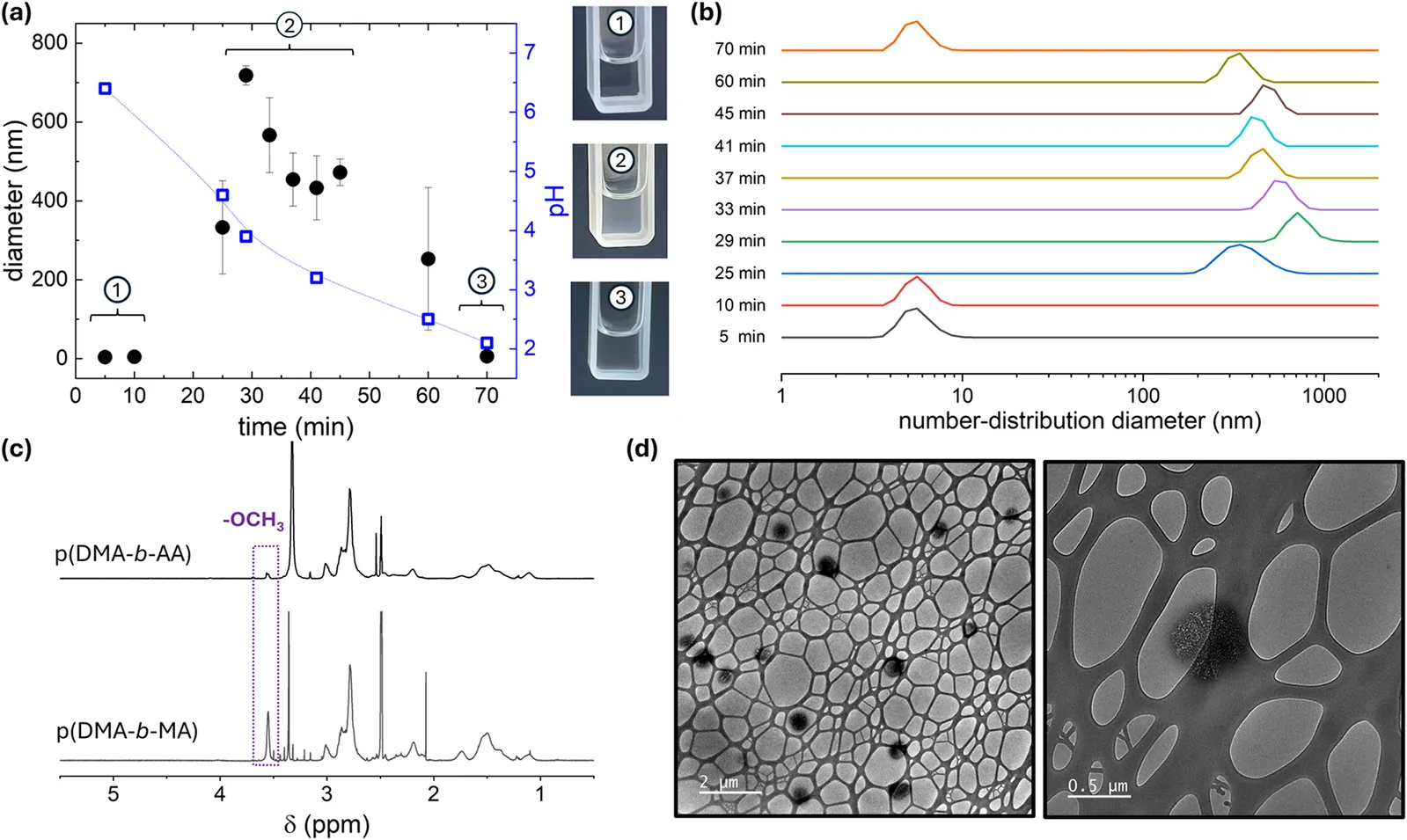
(a) Average particle diameter versus time and (b) number-distribution diameter versus time from DLS during a ct-FISA. (c) ^1^H NMR spectra in d-DMSO of the BCP after ∼30 min. For (a) through (c), ct-FISA was conducted with 0.5 wt % BCP and 5.9 eq DMS in 0.9 M buffer at a pH = 6.4. (d) TEM micrographs of the isolated assemblies were taken using identical ct-FISA conditions as (a–c) but in water (no buffer) to avoid salts in the micrographs.

In the early stages of the ct-FISA process (*t* ≤ 10 min), the BCPs existed primarily as soluble unimers of ∼4 nm in diameter (Figure 2a,b) and the reaction appeared transparent to the eye (see inset image 1 in Figure 2a). However, around ∼25 min, the particle size in DLS began to rapidly increase, reaching a peak diameter of ∼700 nm at 30 min. This transition was visibly reflected in the solution turning cloudy (see inset image 2 in Figure 2a). These results suggest that the BCPs begin to assemble into larger aggregates as the pAA repeat units are progressively converted into pMA repeat units during methylation (Scheme 1i). To validate that methylation was indeed occurring, the reaction was sampled at 30 min for structural analysis with ^1^H NMR. As shown in Figure 2c, a new methyl group resonance was observed at ∼3.6 ppm, characteristic of pMA repeat units,^43,44^ which was absent in the p­(DMA-*b*-AA) precursor before addition of DMS. NMR integration revealed ∼34 of 40 pAA repeat units were converted to pMA repeat units (85% conversion) at the time of sampling. Holistically, this result in combination with DLS confirms that methylation of the carboxylates drives a hydrophilic-to-hydrophobic transition in the BCP, underpinning the FISA mechanism and the transition from unimers to self-assembled particles. Similar to traditional FISA, we suspect that a critical degree of functionalization must be reached to form a sufficiently hydrophobic domain to induce self-assembly,^11^ however, research efforts to accurately quantify this threshold are still ongoing.

After reaching a maximum particle size, the diameter gradually decreased back to unimers from ∼30 to 70 min, marking the completion of the chemically fueled transient self-assembly cycle (Figure 2a,b). This decrease in particle size was accompanied by a visual return to a transparent solution (Figure 2a, inset image 3). At this stage in the ct-FISA process, hydrolysis dominates (Scheme 1ii) and the methyl esters of the hydrophobic pMA block are progressively reverted back to hydrophilic pAA repeat units, eventually causing particle disassembly. We rationalize that hydrolysis begins to govern the system because as the ct-FISA progresses, the pH of the reaction mixture decreases (i.e., ∼6.5 → 2, Figure 2a, blue data) from decomposition of DMS and acidification (see Scheme 1iii),^19^ which slows and eventually stops the methylation reaction.^45,46^ Crucially, when the pH drops significantly below the p*K*_a_ of the pAA repeat units (∼4.5), the active reagent (*the carboxylates*) needed for methylation are no longer available. We note, the point where the particle size begins to decrease coincides directly with when the system’s pH roughly equals the p*K*_a_ of pAA block, possibly representing the tipping point from an *alkylation* dominated system (Scheme 1i) to a *hydrolysis* dominated system (Scheme 1ii). Additional experiments were conducted to rule out any possibility of pH-induced self-assembly from conversion of the carboxylates to carboxylic acids and they showed p­(DMA-*b*-AA) alone, *without* DMS, was incapable of particle formation at pH values from ∼6.0 to 1.5 (Figure S9). The persistence of unimer-sized BCPs across these pH values indicate that the pAA block is *incapable* of inducing self-assembly itself under our conditions, reinforcing the important role of DMS and a chemically fueled methylation in particle formation.

To gain further insights into the particle morphology, an ongoing ct-FISA experiment was sampled for TEM (Figures 2d, S10). Initial attempts were not successful, as the buffer salts contaminated samples and obfuscated images, making visualization of the pure particles challenging. Thus, ct-FISA was conducted in pure water, under otherwise identical conditions. Under these new conditions, it was necessary to sample ct-FISA after ∼2 min for particle observation due to different particle lifetimes and assembly/disassembly kinetics (discussed later). From the TEM micrographs (Figure 2d), spherical particles (∼500–600 nm in diameter) were observed that aligned with DLS, which was consistently observed across all our images (Figure S10). We note, however, that these TEM images support the formation of spherical particles under *aqueous* ct-FISA conditions but may not conclusively represent the morphology formed under standard buffered conditions. Moreover, although TEM and DLS support the formation of large, transient polymer assemblies, the present data does not resolve their internal structure, an important area of future morphological research.

### Impact of pH and Buffer Concentration

To better understand the implications of the reaction media on ct-FISA, a series of experiments were conducted to investigate the initial pH and buffer concentration (Figure 3). Such parameters are important in this aqueous ct-FISA system since they influence many of the involved reactions, reactant speciation, and solvent polarity. Based upon the Henderson–Hasselbalch relationship,^47^ the pAA block begins primarily in its carboxylate form (∼99 to 93%) under the conditions employed in this pH study (∼6.4 to 5.6) respectively. Overall, experimentation with pH revealed that the particle lifetime decreases as the initial system pH decreases (Figure 3a). We define the particle lifetime as the time frame from the onset of assembly until the system returns to unimers. Quantitatively, as the initial pH decreased from 6.4 to 6.0 to 5.6, the particle lifetime was found to decrease from ∼60 to 45 to 35 min, respectively. These decreases in lifetime are a result of shorter times to disassembly. For example, as the pH decreased from 6.4 to 5.6, the time needed for disassembly and reversion to unimers occurred after ∼70 and 40 min, respectively. This behavior makes sense considering that the time available for methylation should decrease as the system’s initial pH is lowered, since the pH will reach the p*K*_a_ of the pAA repeat units sooner and remove the carboxylate reactants needed for methylation earlier. Further decreases of the system’s pH to 5 revealed a boundary condition where self-assembly and particle formation fail to occur (Figure S11), suggesting a nearly complete inhibition of methylation and formation of hydrophobic repeat units.

**Figure 3:**
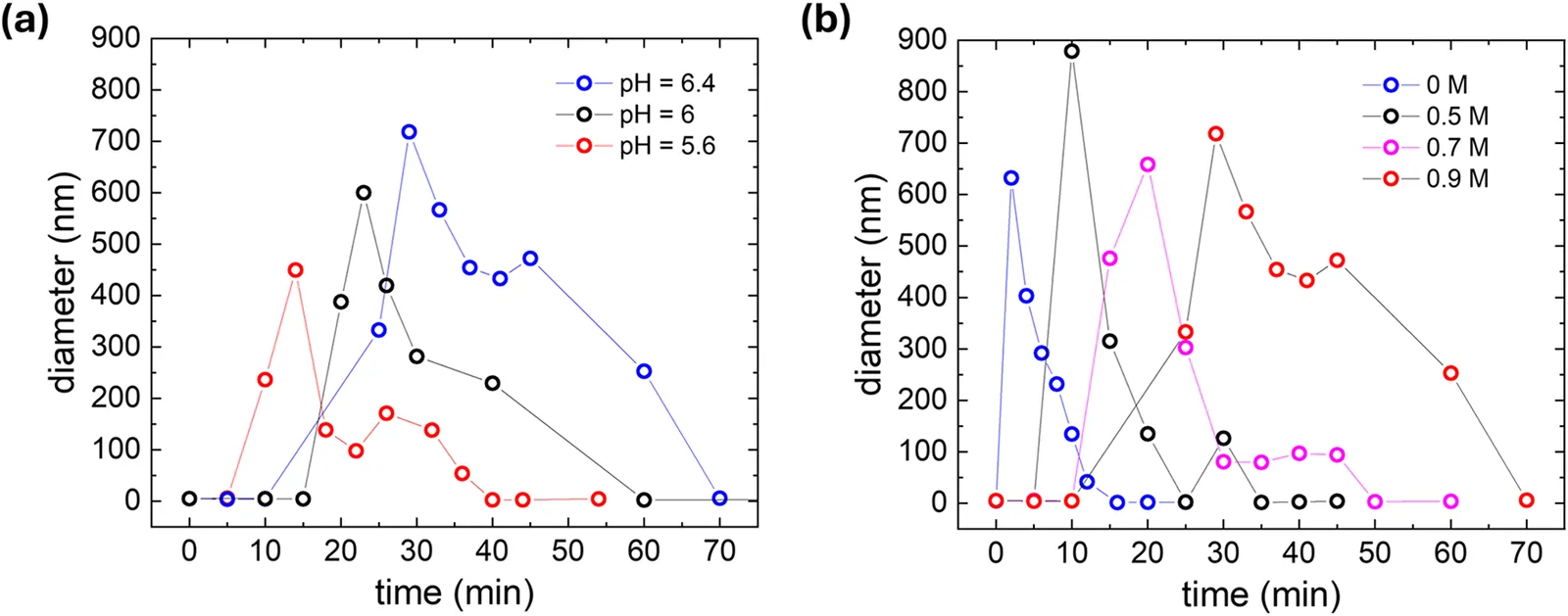
Impact of initial pH and buffer concentration on ct-FISA. (a) Average particle diameter versus time as a function of the initial pH. ct-FISA was conducted with 0.5 wt % BCP and 5.9 eq DMS in 0.9 M buffer. (b) Average particle diameter versus time as a function of the buffer concentration. ct-FISA was conducted with 0.5 wt % BCP and 5.9 eq DMS at a pH = 6.4.

Beyond pH, the buffer concentration was also found to play a critical role in modulating system dynamics (Figure 3b). In general, increasing the buffer concentration from 0 to 0.9 M extended particle lifetime from ∼15–20 min (0–0.5 M) to 40 min (0.7 M) to 60 min (0.9 M). This effect arises due to increased buffering capacity, which slows acidification of reaction media and permits the alkylation reaction to continue for longer durations. Moreover, less concentrated buffers were observed to have earlier onsets and time to peak particle sizes, which is clearly exemplified by the nearly immediate particle formation and peak particle size observed in the system without buffer (pure water). Together, our experiments on buffer and pH, highlight the intricate interplay between the reaction media and the transient behavior of chemically fueled FISA, offering a model self-assembly system that can respond to environmental cues to study kinetics and nonequilibrium assembly/disassembly processes.

### DMS Concentration and Number of Doses (Cycling)

In addition to the reaction media, we wanted to understand how the amount and dosing of fuel impacted the ct-FISA process (Figure 4a). The chemical fuel, or DMS concentration, is a critical parameter driving the methylation of carboxylate groups, and its availability should directly impact the extent of conversion to hydrophobic pMA and particle lifetime. Therefore, a series of ct-FISA experiments were conducted over a range of DMS concentration from [DMA]/[COOH] = 4.4 to 17.7. At low DMS concentrations, particularly when the molar ratio of [DMS]/[COOH] ≤4.4, no self-assembly was observed. Under these low concentrations, we reason insufficient fuel exists to effectively methylate the carboxylate groups, sustain particle formation, and outpace the hydrolysis reaction (Scheme 1ii) because DMS is being consumed to a meaningful extent by the competing reaction with water/hydroxide ions (Scheme 1iii).^19^ In contrast, at intermediate concentrations of DMS, i.e., [DMS]/[COOH] ≈ 5.9–11.8, particles formed robustly, reaching sizes of ∼600–800 nm that persisted for longer lifetimes of 40–60 min due to sufficient levels of fuel being supplied. However, at higher DMS concentrations of [DMS]/[COOH] ≥17.7 (Figures 4a, S12), the particle lifetime began to shorten again (∼30 min) likely due to rapid acidification of the reaction media (Scheme 1iii) and inhibition of the methylation reaction. Higher DMS concentrations could not be explored due the solubility limit of DMS in the reaction media. Overall, these results underscore the critical role of DMS in balancing the system and its downstream effects on assembly, disassembly, and the particle transience.

**Figure 4:**
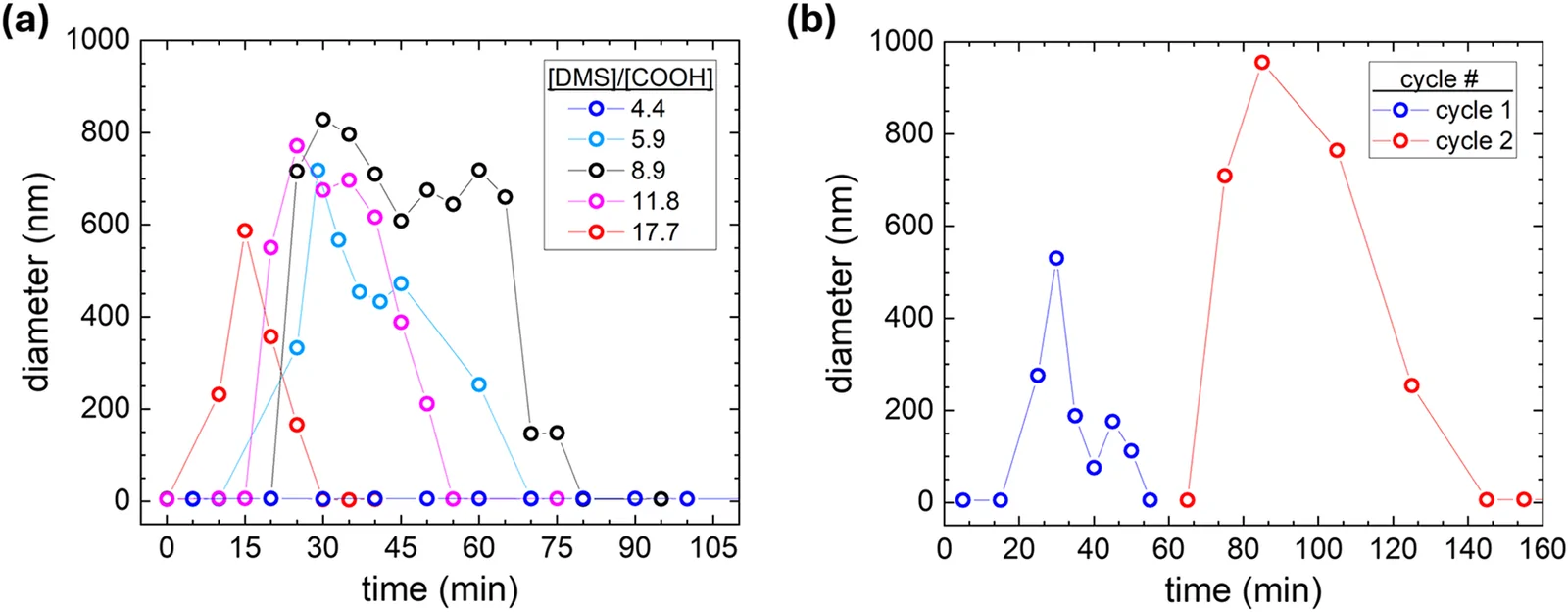
Impact of the DMS concentration and number of DMS doses on ct-FISA. (a) Averaged particle diameter versus time as a function of the DMS concentration or molar ratio of [DMS]/[COOH]. Reactions were conducted with 0.5 wt % BCP in 0.9 M buffer at a pH = 6.4. (b) Averaged particle diameter versus time after two separate injections of 5.9 equiv of DMS. ct-FISA was conducted with 1 wt % BCP in 0.9 M buffer at a pH = 6.4.

Our final experiment with DMS explored multiple doses and the cycling ability of our ct-FISA system (Figure 4b). Cycling, or the capability to undergo repeated assembly/disassembly processes, is an important parameter for understanding a system’s robustness (tolerance to waste or byproducts) and ability to continuously dissipate chemical energy for self-assembly upon fuel replenishment. To investigate, we conducted ct-FISA using 1 wt % BCP in 0.9 M buffer with two sequential doses of DMS at 5.9 equiv each. In the first cycle, upon addition of the first dose of DMS, the BCPs transitioned from unimers to particles and back to unimers similar to other ct-FISA experiments (Figure 4b, blue data). However, initial attempts to reform particles upon addition of a second dose of DMS were unsuccessful, as the pH had dropped to ∼1.5 from decomposition of DMS during the first dose. Fortunately, upon readjusting the pH to back to 6.4 with NaOH, a second cycle could be successfully initiated using the same DMS concentration (Figure 4b, red data). This result highlights the system’s transient and reversible nature (a fundamental aspect of DSA), its ability to tolerate some buildup of chemical waste (e.g., (CH_3_O)­SO_3_^–^, CH_3_OH), which often halt DSA systems,^48^ and its sensitivity to acidity and inability to initiate a second cycle without pH adjustments.

## Conclusion

In summary, we developed a chemically fueled and transient FISA platform in water based on DMS-mediated methylation of p­(DMA-*b*-AA). This system couples a forward alkylation reaction that transiently increases hydrophobicity with a reverse hydrolysis pathway that restores the original hydrophilic polymer, enabling spontaneous assembly and disassembly of BCP particles under ambient conditions. Polymer synthesis and characterization confirmed access to the targeted p­(DMA-*b*-AA) precursor, while DLS, NMR, and TEM demonstrated transient particle formation, transient methyl ester generation, and a spherical aggregated morphology. The lifetime and evolution of the assembled state were governed by the reaction conditions: lower initial pH shortened particle lifetimes, higher buffer concentration prolonged assembly by resisting acidification, and DMS concentration exhibited an optimum window that balanced methylation against competing hydrolysis and pH drop. A second assembly cycle could be induced after pH readjustment, highlighting the reversible and dissipative nature of the system while also revealing its sensitivity to accumulated acidity. Collectively, these results establish ct-FISA as a versatile nonequilibrium self-assembly strategy that expands the chemistry available for transient BCP structures and provides a foundation for future studies on tunable lifetimes, morphological control, and chemically responsive materials.

## Supplementary Material



## Notes

Additional experimental characterization data, including NMR and FTIR spectra of synthesized polymers; representative DLS intensity, volume, and number distributions; pH-control DLS experiment without DMS; TEM images of transient assemblies; and additional DLS experiments showing effects of initial pH and DMS concentration (PDF)
