# Supplementary material for: Chemically Fueled Functionalization-Induced Self-Assembly of Block Copolymers for Transient Assemblies in Water
Source: ACS Polym Au. 2026 Jun 18;6(5):1354–62. doi: 10.1021/acspolymersau.6c00089 (PMC13673880; doi:10.1021/acspolymersau.6c00089)
Supplement: Supplementary file 1 [file lg6c00089_si_001.pdf]

Supporting Information

Chemically-Fueled Functionalization-Induced Self-Assembly of Block Copolymers for Transient Assemblies in Water

*Zehou You and Andrew J. D. Magenau**

Department of Materials Science & Engineering, Drexel University, Philadelphia, Pennsylvania 19104, United States

*Corresponding author: ajm496@drexel.edu

CONTENTS

Fig. S1 2

Fig. S2 3

Fig. S3 4

Fig. S4 5

Fig. S5 6

Fig. S6 7

Fig. S7 8

Fig. S8 9

Fig. S9 10

Fig. S10 11

Fig. S11 12

Fig. S12 13

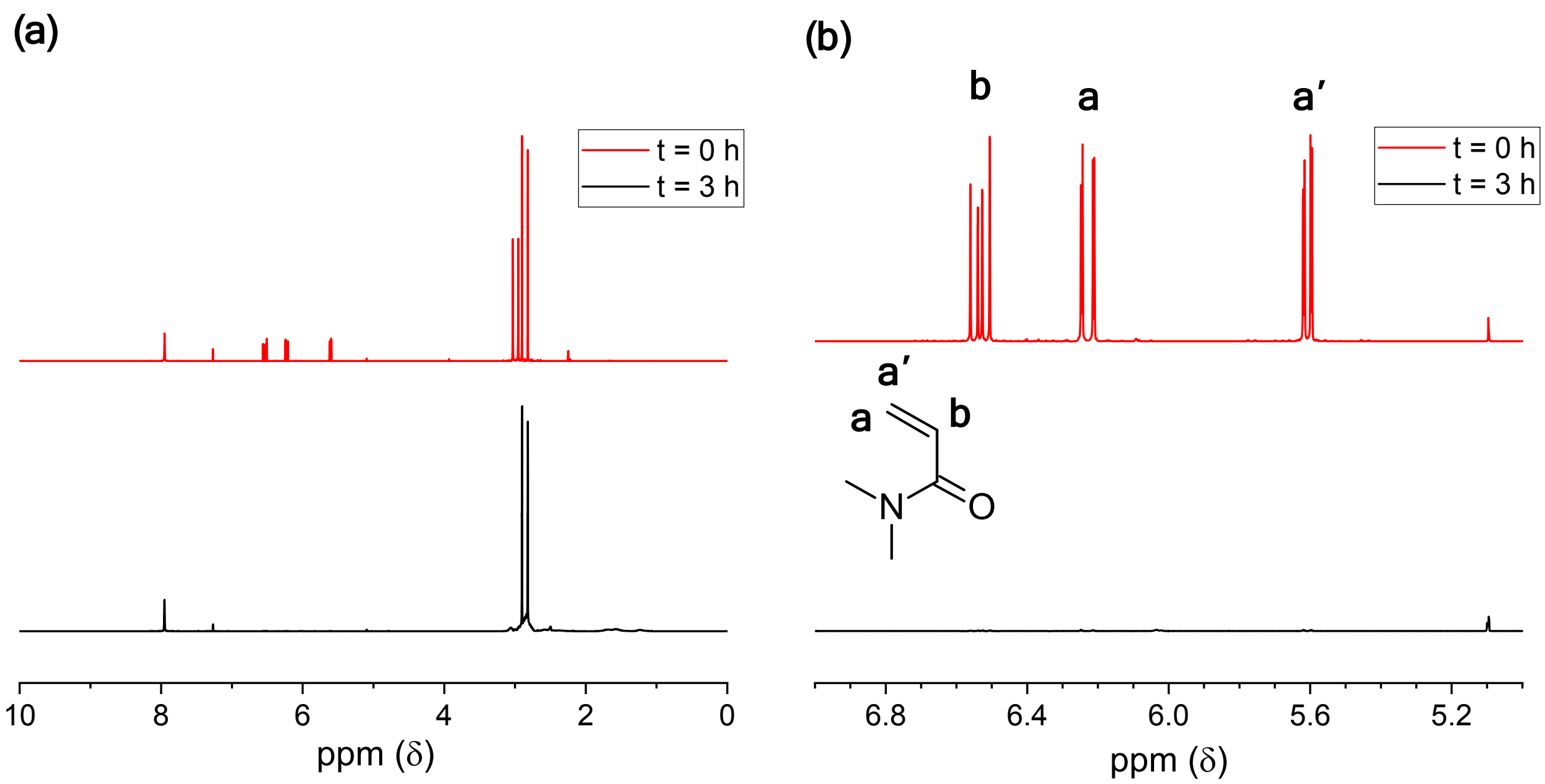

Fig. S1. ^{1}H NMR in $CDCl_3$ of a RAFT polymerization of DMA at the initial and final stages of polymerization. Figure (a) shows the full proton NMR and figure (b) shows a zoomed-in spectra of the vinylic region with near quantitative conversion of DMA. Trioxane was used as an internal standard for monomer conversion calculations.

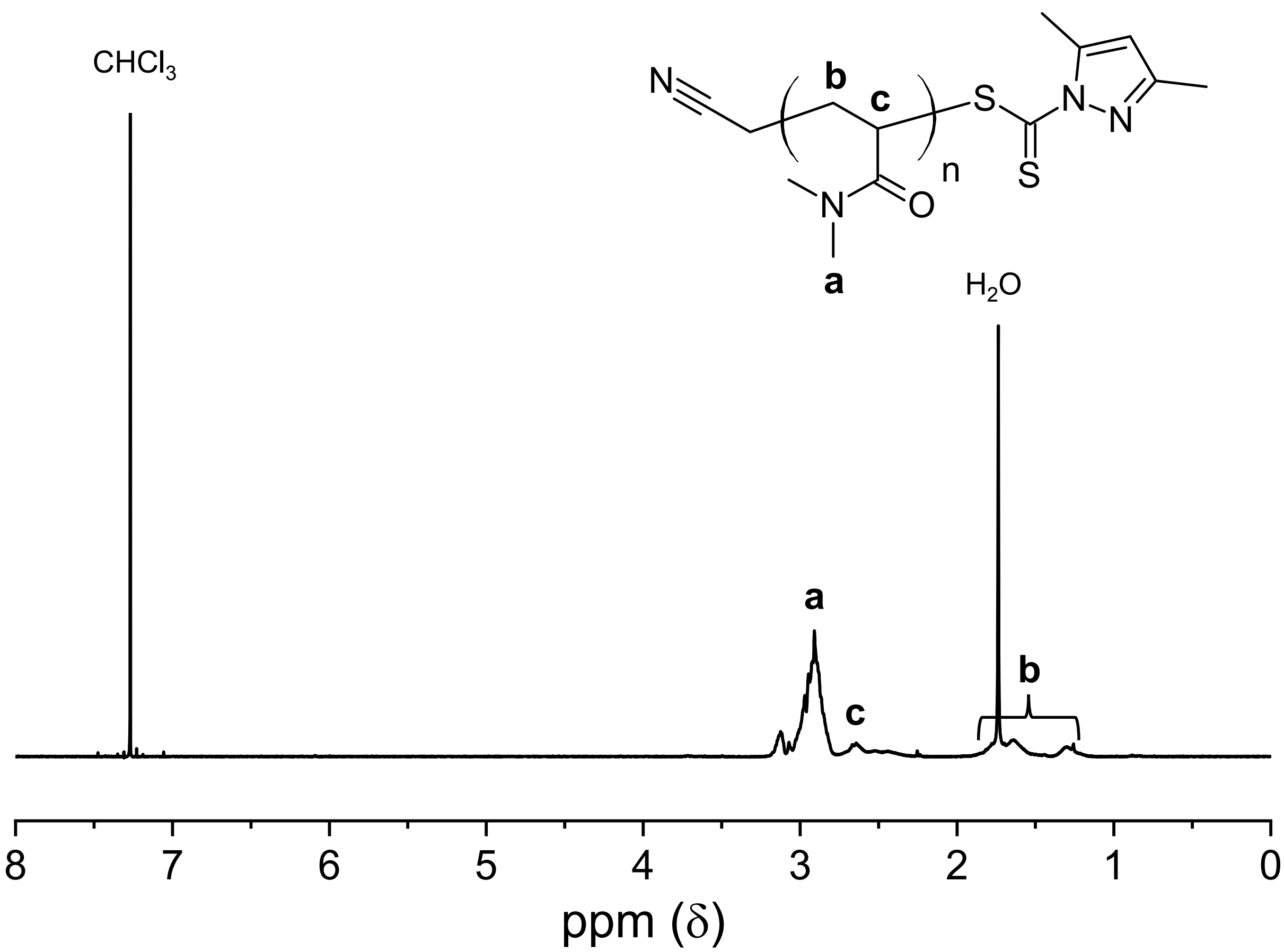

Fig. S2. ^{1}H NMR of p(DMA) macro-CTA in $CDCl_3$.

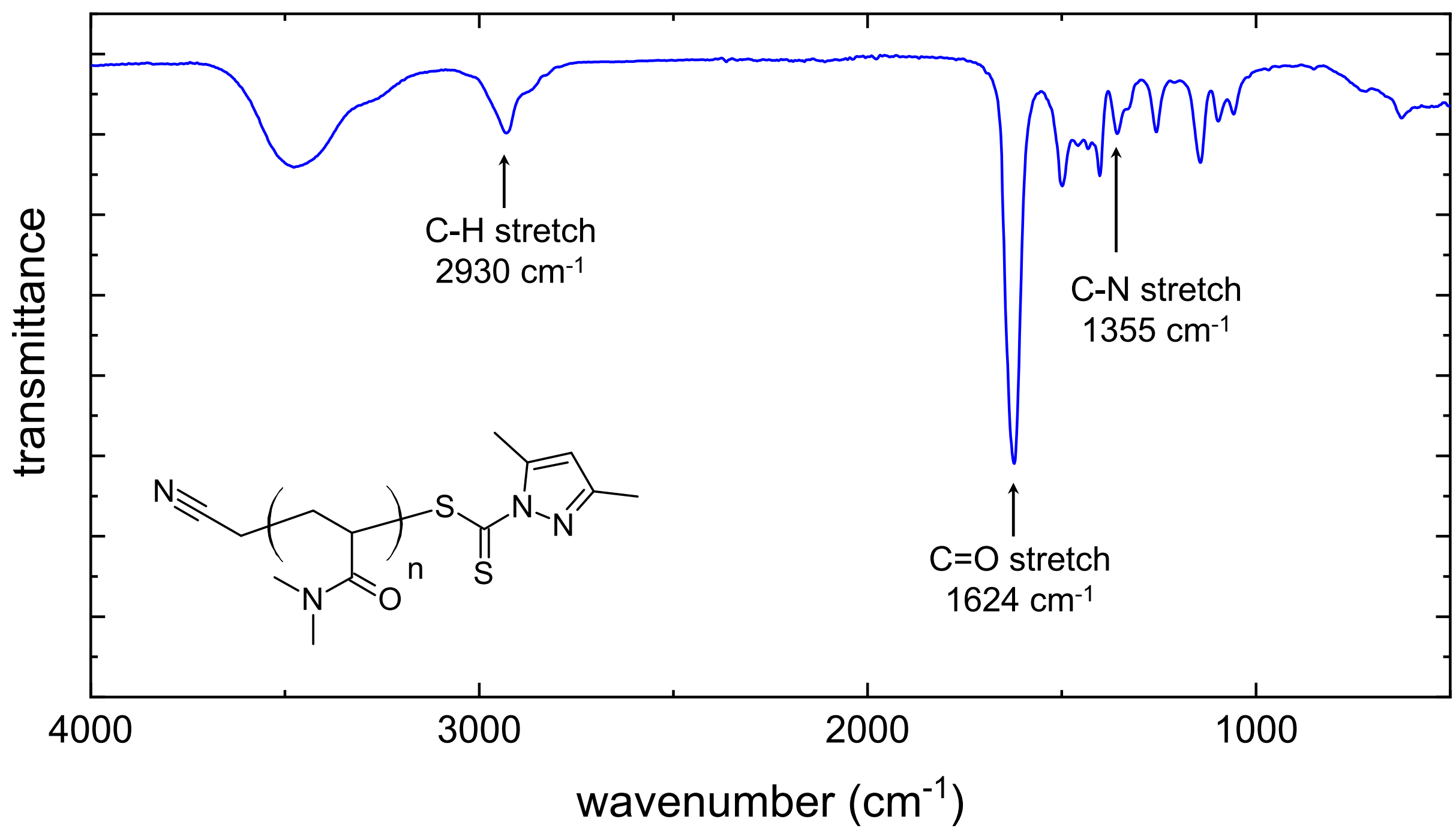

Fig. S3. FTIR spectrum of p(DMA) macro-CTA with band assignments.

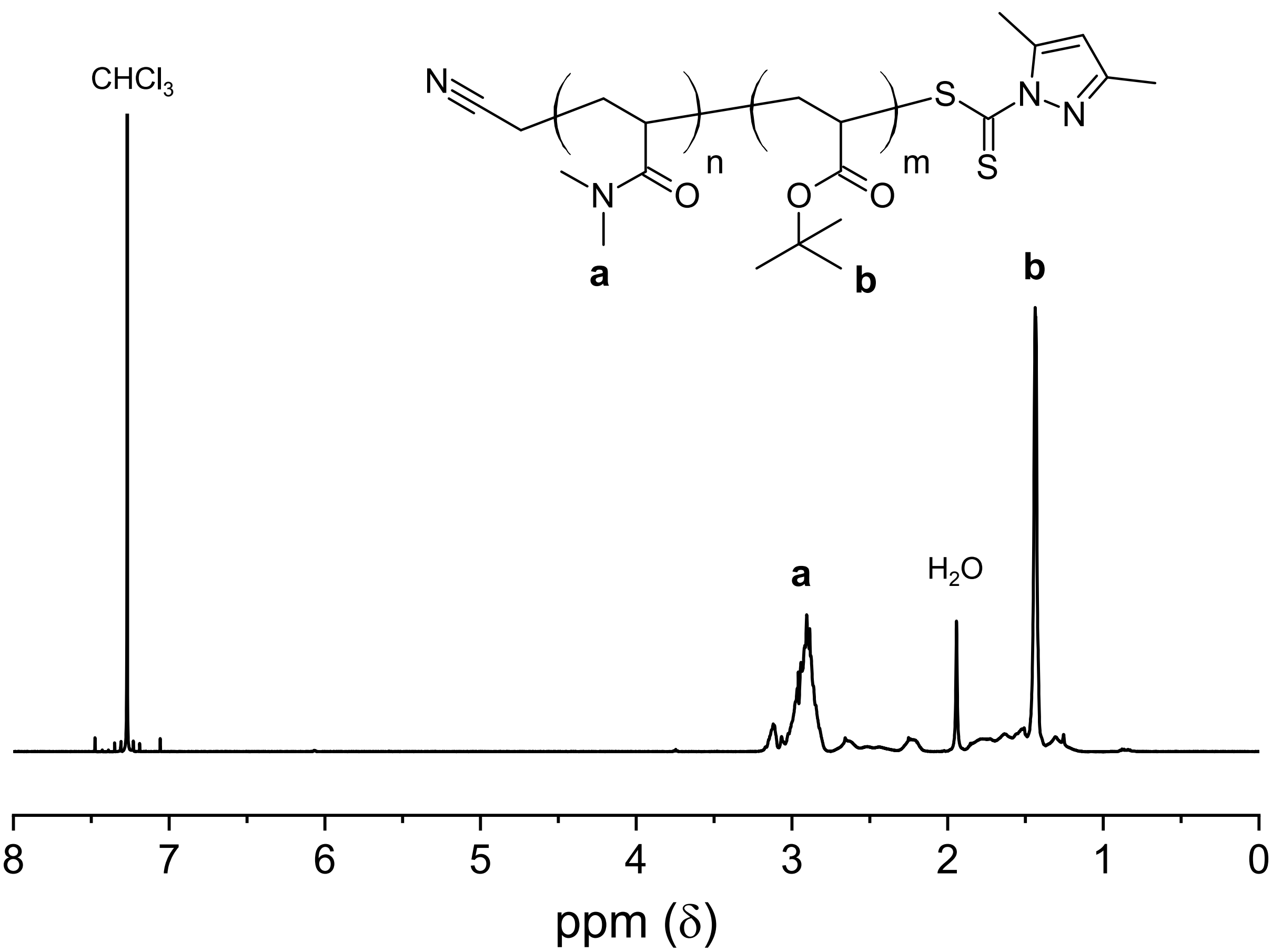

Fig. S4. ^{1}H NMR spectrum of p(DMA-*b*-tBA) in $CDCl_3$.

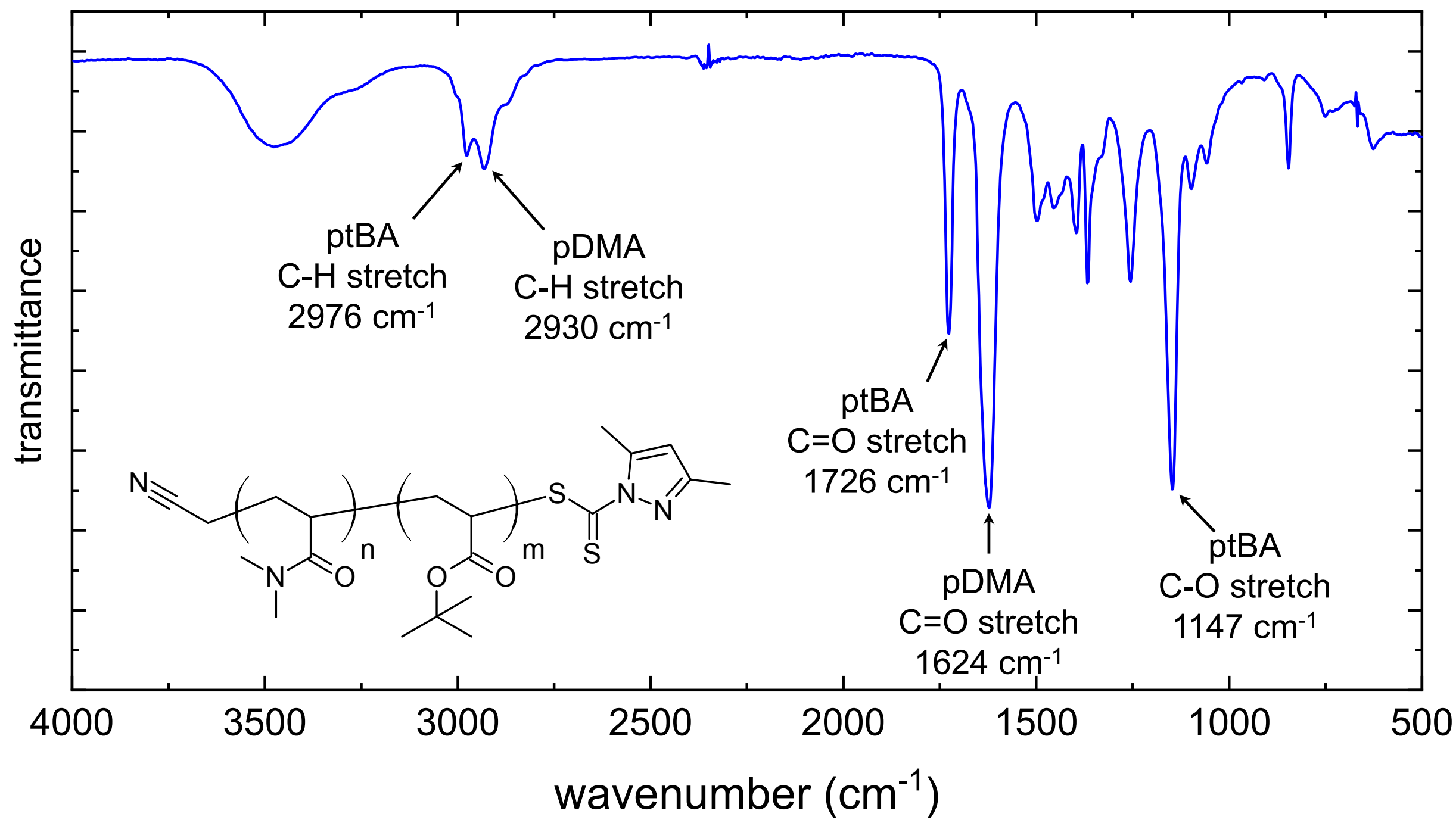

Fig. S5. FTIR spectrum of p(DMA-*b*-tBA) with band assignments.

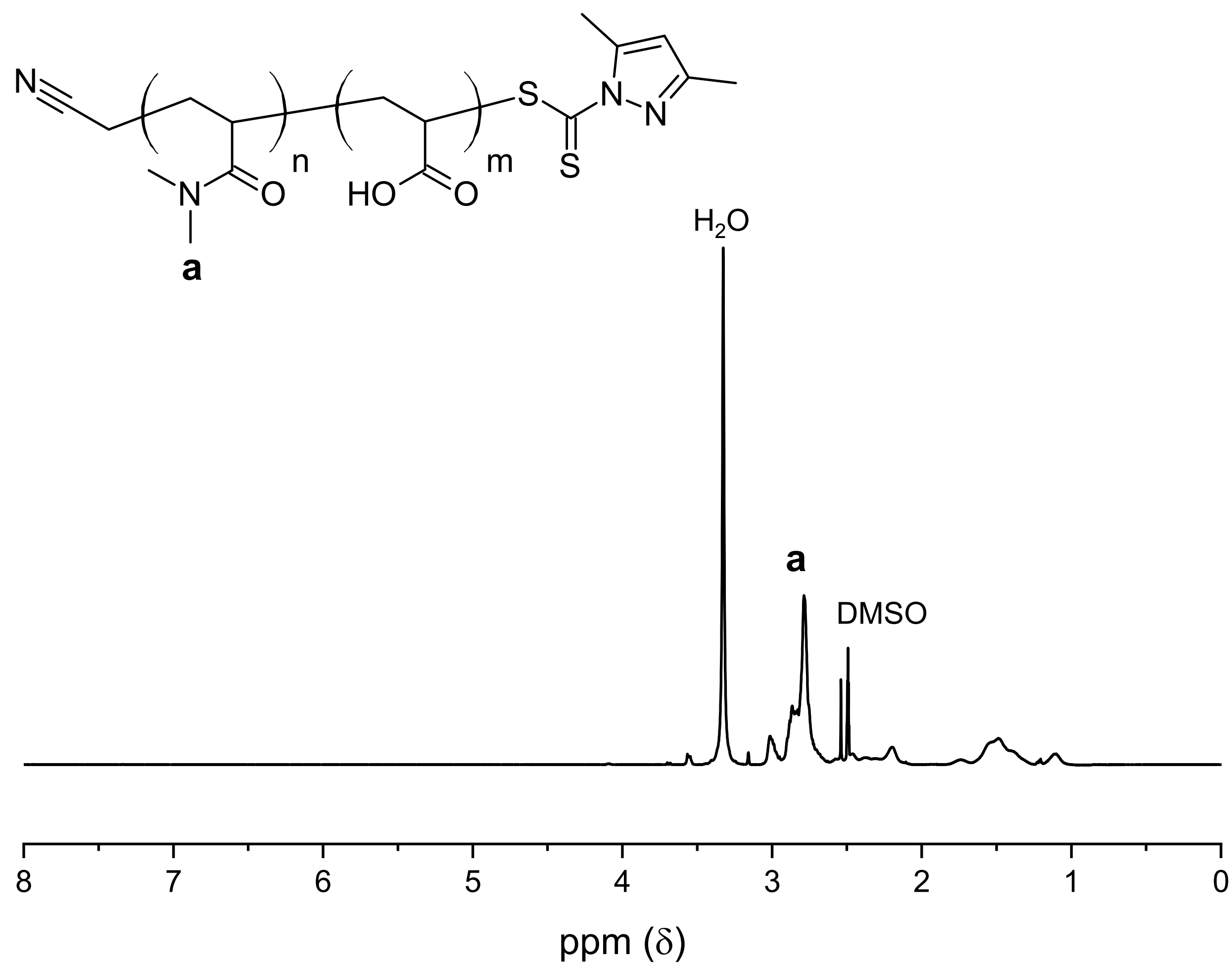

Fig. S6. ^{1}H NMR spectrum of p(DMA-*b*-AA) in d-DMSO.

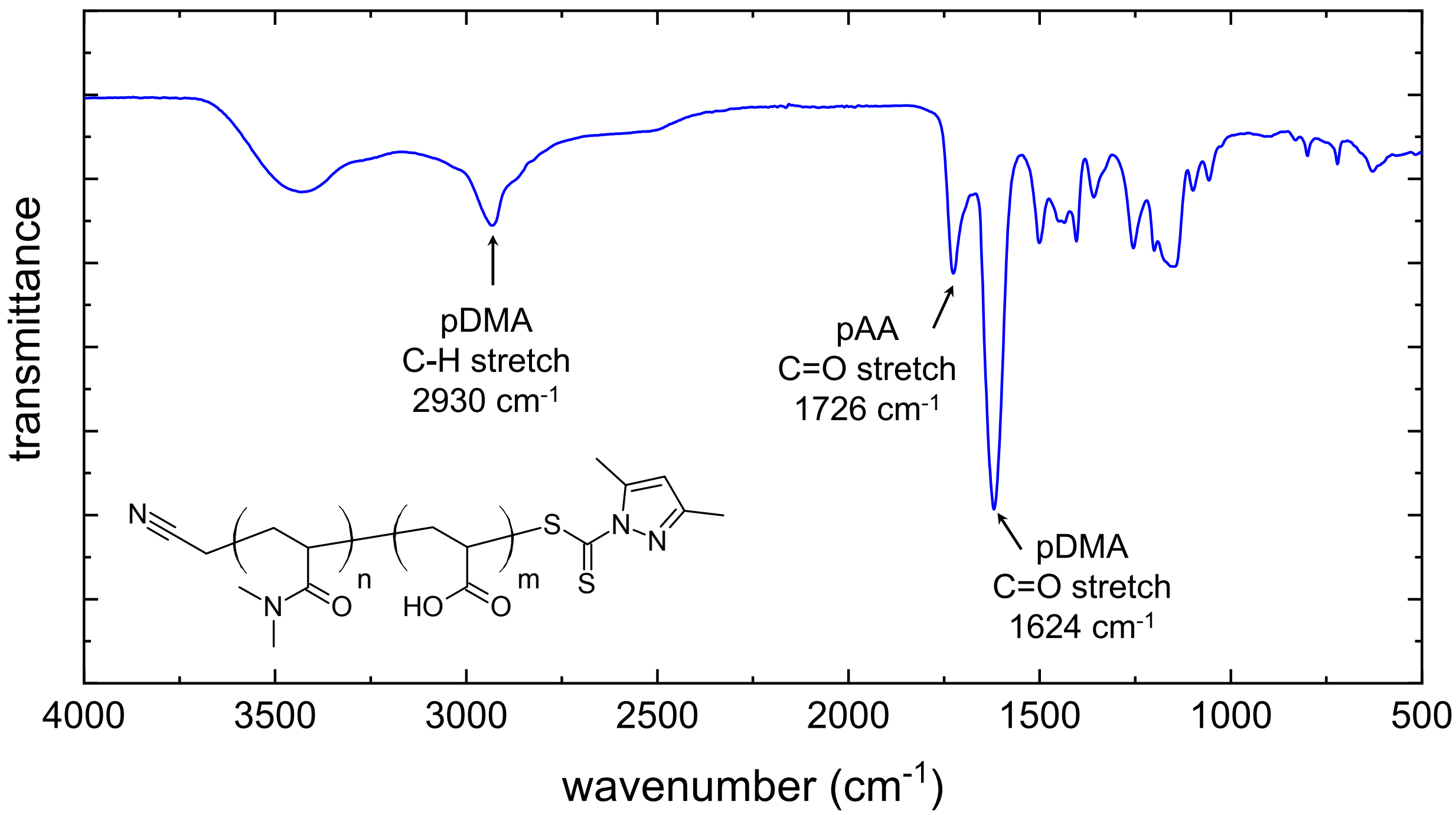

Fig. S7. FTIR spectrum of p(DMA-*b*-AA) with band assignments.

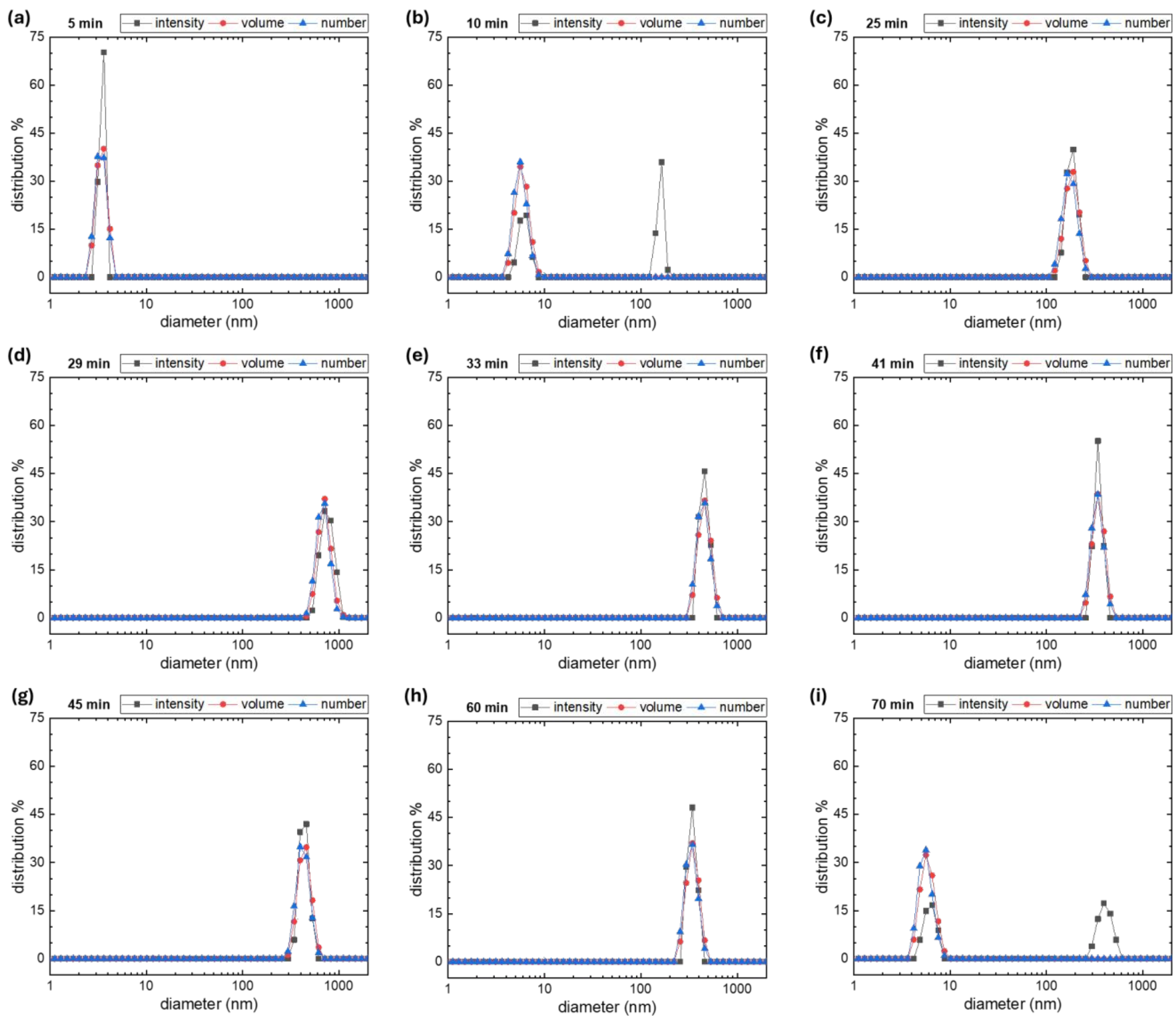

Fig. S8. Representative intensity, volume, number DLS distribution for ct-FISA with 0.5 wt % p(DMA_{100}-*b*-AA_{40}) in 0.9 M buffer after addition of 5.9 equivalents DMS. Each data point reported in Fig.2(a) is an average of three separate measurements.

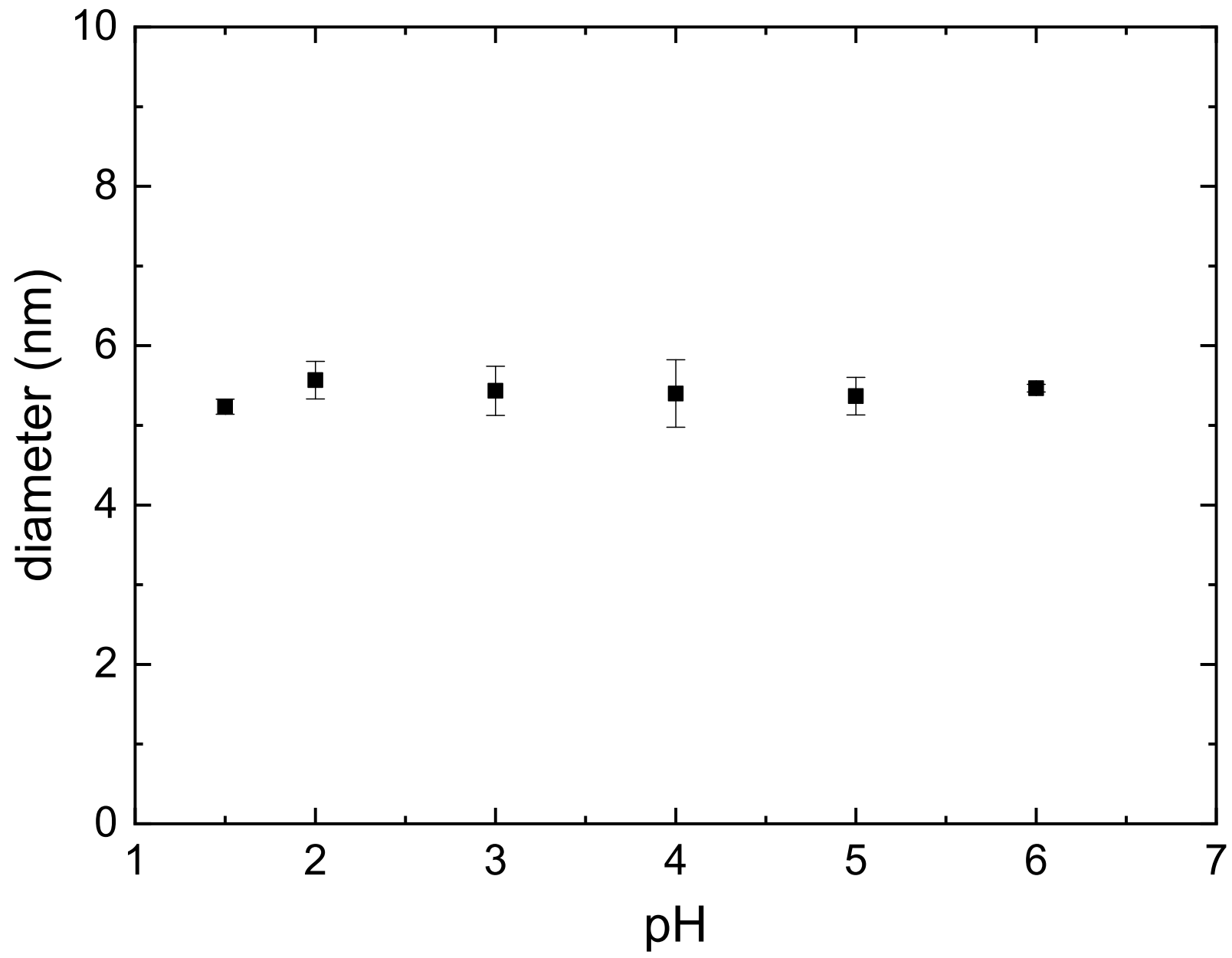

Fig. S9. Number average diameter from DLS of 0.5 wt.% p(DMA_{100}-*b*-PAA_{40}) in 0.5 M buffer at different pH values without DMS.

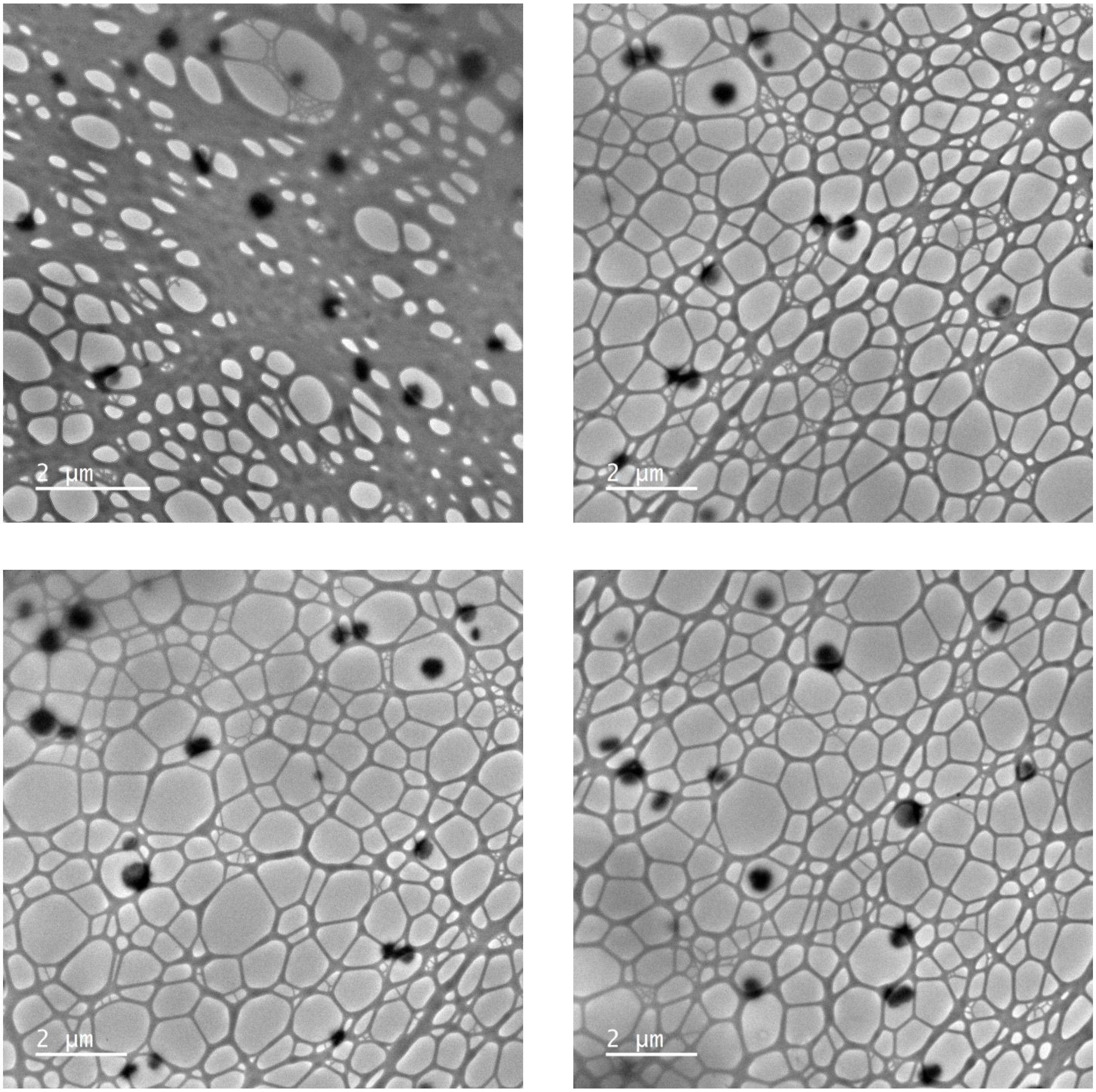

Fig. S10. TEM images of ct-FISA particles. ct-FISA was conducted with 0.5 wt.% BCP and 5.9 equivalents of DMS in pure water at a pH = 6.4. The experiment was sampled at ~2 min after DMS addition since the particle lifetime is shorter in pure water.

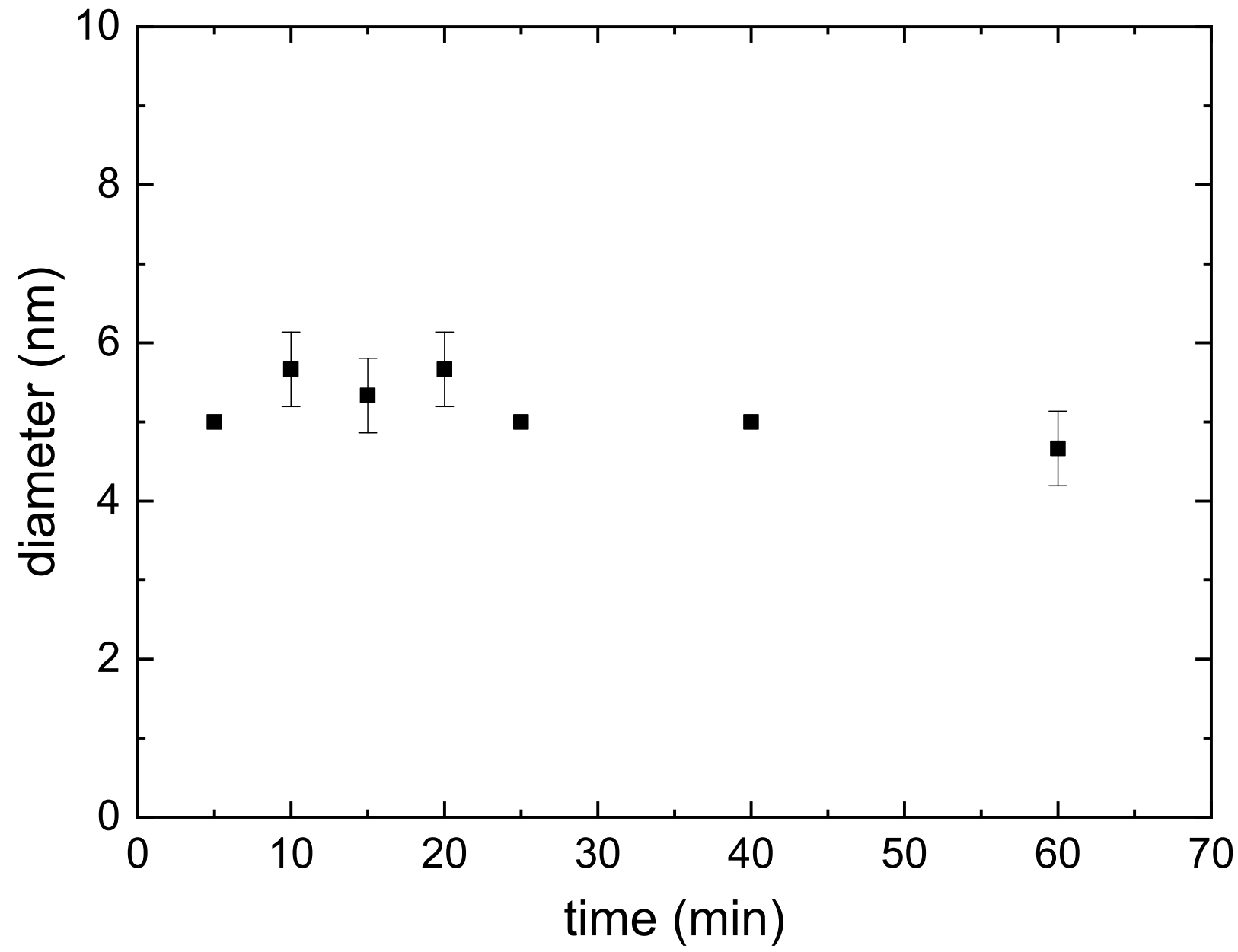

Fig. S11. Particle diameter from DLS as a function of time for ct-FISA with 0.5 wt % p(DMA_{100}-*b*-AA_{40}) in 0.9 M buffer at a pH = 5 with 5.9 equivalents DMS.

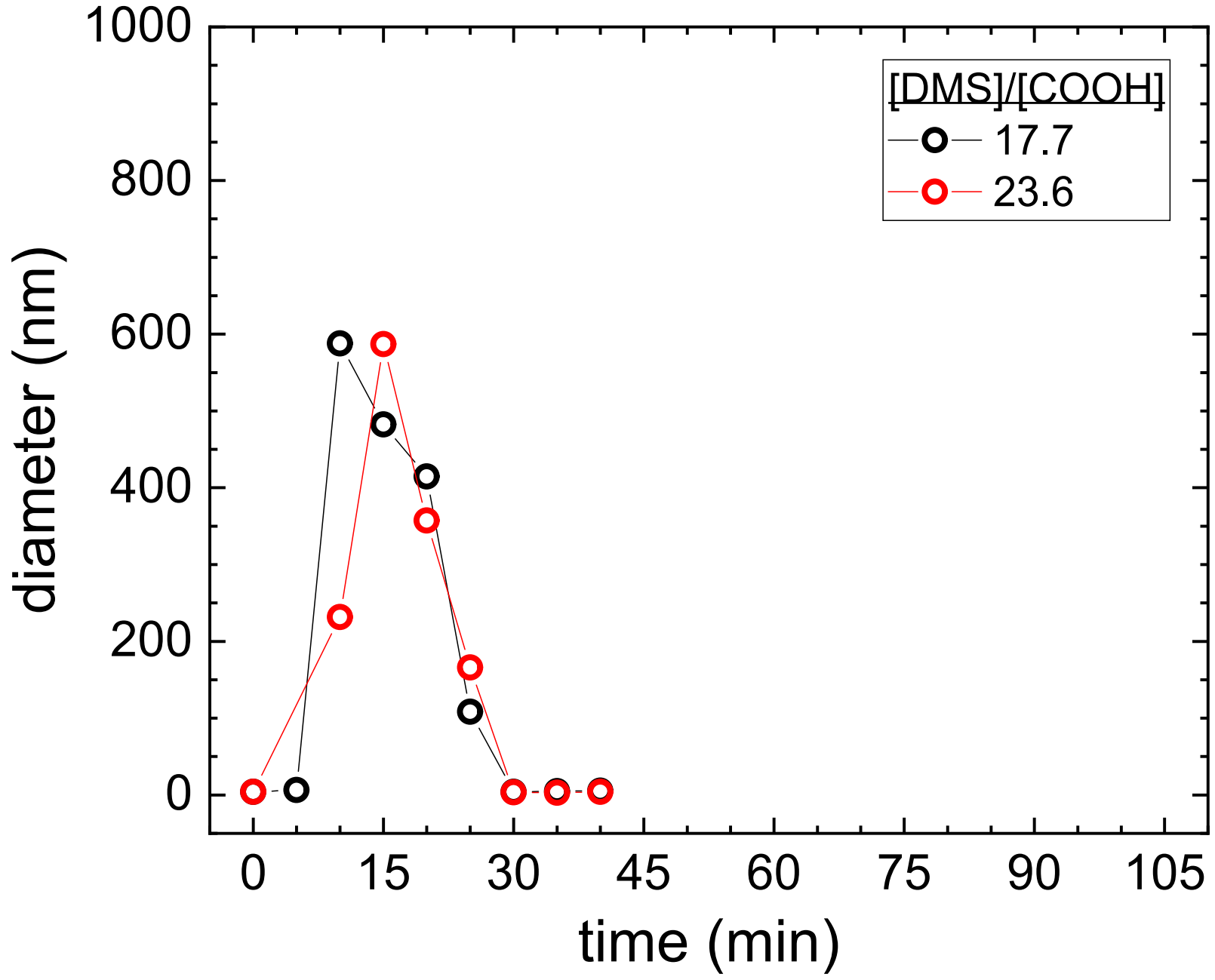

Fig. S12. Particle diameter from DLS as a function of time for ct-FISA with 0.5 wt % p(DMA_{100}-*b*-AA_{40}) in 0.9 M buffer at a pH = 6.4.
